# The complete mitochondrial genome of *Megalurothrips usitatus* (Bagnall 1913) (Thysanoptera: Thripidae) and its phylogenetic analysis

**DOI:** 10.1080/23802359.2022.2089065

**Published:** 2022-06-28

**Authors:** Jiang-Hui Cheng, Xiao-Wei Li, Li-Min Chen, Hai-Bin Han, Yao-Bin Lu, Yong-Ming Ruan

**Affiliations:** a College of Chemistry and Life Science, Zhejiang Normal University, Jinhua, Zhejiang, China; b State Key Laboratory for Managing Biotic and Chemical Threats to the Quality and Safety of Agro-products, Institute of Plant Protection and Microbiology, Zhejiang Academy of Agricultural Sciences, Hangzhou, Zhejiang, China; c Institute of Grassland Research, Chinese Academy of Agricultural Sciences, Huhhot, Inner Mongolia, Autonomous Region, China

**Keywords:** *Megalurothrips usitatus*, complete mitochondrial genome, phylogeny, Thysanoptera

## Abstract

*Megalurothrips usitatus* is a serious pest on *Vigna unguiculata*. In this study, the complete mitochondrial genome sequence of *M. usitatus* was characterized and its phylogenetic relationship within the Order Thysanoptera was determined. The mitochondrial genome of *M. usitatus* was a circular molecule of 15426 bp in length, containing 13 protein-coding genes, 2 rRNA genes, 22 tRNA genes, and the control region. It showed the typical insect mitochondrial genome arrangement. The AT content of the whole genome was 77.69% and the length of the control region was 567 bp with 78.66% AT content. The Maximum likelihood (ML) phylogenetic analysis based on mitochondrial protein-coding genes of 17 insect speciesshowed that *M. usitatus* is closest to *Frankliniella occidentalis*.

*Megalurothrips usitatus* (Bagnall 1913) (Thysanoptera: Thripidae) is an important leguminous pest in South China, especially in Hainan province, and has caused enormous losses to cowpea planting industry (Huang et al. 2018). It has been reported that *M. usitatus* is widely distributed, mainly occurs in China, Japan, India, Nigeria, Philippines, etc. ( Han 1997; Dialoke and Bosah 2015; Iftikhar et al. 2016). *Megalurothrips usitatus* has a wide host range, including 49 plant species in 12 families, of which 32 species are leguminous plants. Up to now, there are over 500 species of Thysanoptera described in China (Dang and Qiao 2012). In 2003, Shao and Barker reported the first complete mitochondrial genome sequence in Thysanoptera (*Thrips imaginis*) (Shao and Barker 2003). So far, there are only 17 mitochondrial genomes from Thysanoptera reported in GenBank database, which is far behind Hemiptera and Orthoptera. In this study, the mitochondrial genome of *M. usitatus* was successfully assembled and annotated, and the phylogenetic relationship of *M. usitatu*s with closely related species was determined.

Adults of *M. usitatus* were collected from *Vigna unguiculata* in Chengmai County (N19°44′23.82″, E110°0′7.56″), Hainan Province, China, and preserved in pure ethanol. The collected samples were identified and stored at −40 °C in the Institute of Plant Protection and Microbiology, Zhejiang Academy of Agricultural Sciences, Hangzhou, China (http://www.zaas.ac.cn/, Xiao-wei Li, lixiaowei1005@163.com) under the voucher number MU20180705HN-2. Total genomic DNA was extracted by using a modified cetyltrimethylammonium bromide (CTAB) method (Doyle and Doyle 1987) and applied to 500 bp paired-end library construction using the NEBNext Ultra DNA Library Prep Kit for Illumina sequencing. Sequencing was carried out on the Illumina NovaSeq 6000 platform (BIOZERON Co., Ltd, Shanghai, China). A total of 8.142 Gb of raw reads were generated, and by employing the tool SOAPnuke (v1.3.0) (Chen et al. 2018), the reads having adapter contamination and those with more than 5% unknown bases were removed to obtain clean reads. De novo genome assembly and annotation were conducted by NOVOPlasty (Dierckxsens et al. 2017) and GeSeq (Tillich et al. 2017), respectively. The genomic sequence has been deposited in GenBank with an accession number OK564665.

The complete mitochondrial genome of *M. usitatus* was a typical circular DNA molecule of 15426 bp in length. A total of 37 genes were annotated, including 13 protein-coding genes (PCGs), 22 transfer RNAs (tRNAs), 2 ribosomal RNAs (rRNAs). The AT content of the whole genome was 77.69% and the length of the control region was 567 bp with 78.66% AT content. All 13 PCGs began with ATN (N represents A, T, G, and C) as the start codon. The ATP6, ATP8, COI, COII, COIII, ND3, ND4L and ND6 genes were terminated with TAA as the stop codon, the gene Cyt b ended with the unfrequent stop codon TAG, and the ND5 concluded with the extraordinary stop codon AGA, whereas the other PCGs (ND1, ND2, and ND4) ended with the incomplete stop codon TA or T.

To reveal the phylogenetic relationship of *M. usitatus* with other members in Thysanoptera, phylogenetic analysis was performed based on mitochondrial protein-coding genes of 17 insect species, of which 6 species, *Empoasca flavescens*, *Apolygus lucorum*, *Menochilus sexmaculata*, *Spodoptera frugiperda*, *Tuta absoluta* and *Solenopsis invicta* were served as outgroups. The sequences were aligned by MAFFT v7.309 (Katoh and Standley 2013). The maximum likelihood (ML) bootstrap analysis with 1000 replicates was performed using RaxML v8.2.12 (Stamatakis 2014). The phylogenetic tree showed that *M. usitatus* was closely related to *Frankliniella occidentalis* (Figure 1) and Thripidae and Phlaeothripidae was monophyletic groups (Figure 1), which is consistent with the results of Zhang et al. (2019). Meanwhile, *Megalurothrips* had a close relationship with *Frankliniella* in Thripidae. The genome sequence of *M. usitatus* in this study might provide useful information for Thysanoptera researches.

**Figure 1.**
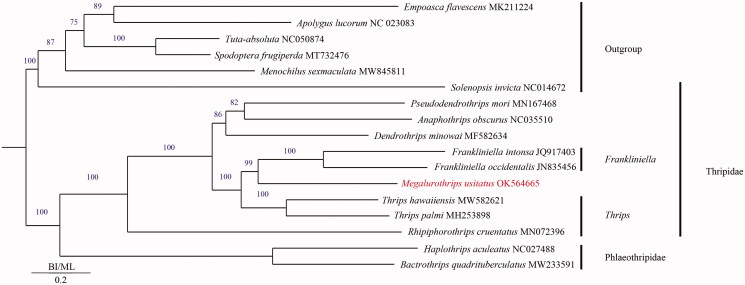
Phylogenetic tree of 17 insect species, including *M. usitatus* based on the nucleotide dataset of the 17 mitochondrial protein-coding genes. The maximum-likelihood bootstrap values are indicated above nodes. The GenBank numbers and tribe of all species are shown in the figure.

## Ethical approval

This study was approved by the Institutional Review Board (IRB) Institutional of Plant Protection and Microbiology, Zhejiang Academy of Agricultural Sciences. The approval code is 2021071700. The approval date is July 17, 2021.

## Author contributions

X-W L, Y-B L planned and designed the research. L-M C, Y-M R collected the insect materials, J-H C performed experiments, and H-B H analyzed the data. J-H C wrote the manuscript.

## Data Availability

The genome sequence data that support the findings of this study are openly available in GenBank of NCBI at (https://www.ncbi.nlm.nih.gov/) under the accession no. OK564665.The associated **BioProject**, **SRA**, and **Bio-Sample** numbers are PRJNA769828, SRR16585084, and SAMN22589897, respectively.
